# Bioinformatic Analysis Reveals High Diversity of Bacterial Genes for Laccase-Like Enzymes

**DOI:** 10.1371/journal.pone.0025724

**Published:** 2011-10-12

**Authors:** Luka Ausec, Martha Zakrzewski, Alexander Goesmann, Andreas Schlüter, Ines Mandic-Mulec

**Affiliations:** 1 Department for Food Science and Technology Biotechnical Faculty, University of Ljubljana, Ljubljana, Slovenia; 2 Center for Biotechnology (CeBiTec), Institute for Bioinformatics, Bielefeld University, Bielefeld Germany; 3 Center for Biotechnology (CeBiTec), Institute for Genome Research and Systems Biology, Bielefeld University, Bielefeld, Germany; Université Paris-Sud, France

## Abstract

Fungal laccases have been used in various fields ranging from processes in wood and paper industries to environmental applications. Although a few bacterial laccases have been characterized in recent years, prokaryotes have largely been neglected as a source of novel enzymes, in part due to the lack of knowledge about the diversity and distribution of laccases within Bacteria. In this work genes for laccase-like enzymes were searched for in over 2,200 complete and draft bacterial genomes and four metagenomic datasets, using the custom profile Hidden Markov Models for two- and three- domain laccases. More than 1,200 putative genes for laccase-like enzymes were retrieved from chromosomes and plasmids of diverse bacteria. In 76% of the genes, signal peptides were predicted, indicating that these bacterial laccases may be exported from the cytoplasm, which contrasts with the current belief. Moreover, several examples of putatively horizontally transferred bacterial laccase genes were described. Many metagenomic sequences encoding fragments of laccase-like enzymes could not be phylogenetically assigned, indicating considerable novelty. Laccase-like genes were also found in anaerobic bacteria, autotrophs and alkaliphiles, thus opening new hypotheses regarding their ecological functions. Bacteria identified as carrying laccase genes represent potential sources for future biotechnological applications.

## Introduction

Laccases are members of the multi-copper oxidoreductases that oxidize a variety of phenolic substances including polyaromatic hydrocarbons (PAH), estrogens in wastewater [1], [2] and recalcitrant biopolymers such as lignin [3], [4]. Due to their broad substrate specificity laccases are of great industrial interest and have been used in paper and wood processing and in the textile industry [5], [6], [7].

Substrate oxidation by laccases (and subsequent reduction of molecular oxygen) creates reactive radicals which can participate in (i) polymerization (oxidative coupling of monomers), (ii) degradation of polymers or (iii) degradation of phenolics (by cleavage of aromatic rings) [8]. Substrate specificity is broadened by mediators, which are small molecular-mass compounds that are oxidized into radicals by laccases and can subsequently oxidize a variety of other (more complex) substrates such as lignin. Laccases contain four copper atoms held in place in the reaction center by conserved copper-binding regions. Nucleotide sequences specifying the copper-binding sites are suitable for molecular-ecological studies as it is possible to design PCR-primers for these sites [9]-[11]. Laccases have been found in all domains of life [12] but have been most intensively studied in ligninolytic fungi [13].

The first indication that laccases may be present in bacteria was based on the phenol-oxidase activity observed in *Azospirillum lipoferum* almost 20 years ago [14]. A decade ago, researchers used the BLAST algorithm to find 14 bacterial laccase genes similar to those known from fungi [15]. A few bacterial laccases have been studied since (see [16] for a more recent review). Until recently, fungal laccases have been considered extracellular enzymes while bacterial laccases were assumed to be mostly intracellular or spore-bound. It was speculated [16] that bacteria may have strategies such as rearrangement of the electron transport system to cope with the toxic molecular compounds produced by the oxidation of aromatic substrates within the cell. The simplistic view of fungal laccases as extracellular lignin-degrading enzymes has given way to a more realistic view, in which fungal laccases are involved in various intra- and extracellular developmental processes in morphogenesis and pathogenesis [7], [12], [17] in addition to their role in degradation of complex substrates. It was suggested that further studies are needed to verify that the diverse fungal laccases retrieved from different environmental studies are indeed extracellular ligninolytic enzymes [3].

In bacteria, the perceived role of laccases has mostly been limited to oxidation of metals and pigment formation [8], [16]. The latter function is based on the well studied CotA laccase located in the spore coat of *Bacillus subtilis*, which produces a melanin-like pigment for the protection of the spore against UV-light [18]. The possibility that bacterial laccases play a role in the degradation of recalcitrant biopolymers has been suggested only recently [4], [19]. However, bacterial laccases may have several properties that are not characteristic of fungal enzymes. Firstly, the laccase from *Streptomyces lavendulae* [20] shows high thermo resistance and the CotA laccase from *Bacillus subtilis* has a half-life of inactivation at 80°C of about 4 h and 2 h for the coat-associated or the purified enzyme, respectively [18]. The most termophilic laccase from *Thermus termophilus* has the optimal reaction temperature of 92°C and a half life of inactivation at 80°C of over 14 hours [21]. Secondly, the laccase from *Bacillus halodurans* is stimulated rather than inhibited by chloride [22], which is a novel trait of great importance for industrial processes. Thirdly, but perhaps most importantly, several pH-tolerant bacterial laccases with pH ranges from 4 up to 9.5 have been described, e. g. from a *Gammaproteobacterium* [23], *Streptomyces* [24], [25], *Bacillus halodurans* [22], and metagenomic sources [26]. The heterologous expression of bacterial laccases may be more efficient than that of fungal laccases as there are no introns or post-translational modification (fungal laccases are glycosylated). Finally, a novel evolutionary lineage of two-domain laccases has been established [27]. These laccases are different from the well-known monomeric three-domain laccases that are typical for fungi and bacteria. The two-domain laccases, which have only been identified in prokaryotes, have a homotrimeric quaternary structure and form the active site on the interface of each two monomers. Three groups of two-domain laccases were distinguished on the basis of the organization of the copper-binding regions within the protein domains, and representative enzymes of type B and type C two-domain laccases have subsequently been characterized in bacteria, while sequence data suggested the presence of type A two-domain laccases in archaea [27]. For all these reasons, studying bacterial laccases is important from the perspectives of basic science as well as for the development of novel biotechnological applications.

The aim of this study was to use the extensive sequence data of the complete and draft bacterial genomes to evaluate bacterial laccases at the level of (1) the distribution of laccase-like genes within different bacterial phyla, (2) the diversity of the genes for bacterial laccases, and (3) the structural characteristics of the putative laccases. The bioinformatic search for new genes was based on profile Hidden Markov Models (pHMMs). This approach provided the theoretical ground for new hypotheses about the roles of laccases in bacteria and may guide the future research of these interesting and biotechnologically important enzymes.

## Methods

### Construction of profile Hidden Markov models (pHMMs)

**Figure 1:**
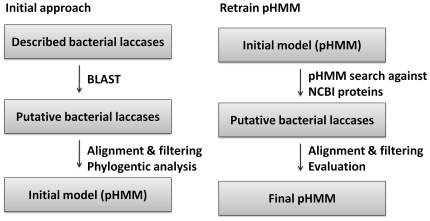
A two-step approach for the construction of laccase-specific profile Hidden Markov Models (pHMM). An initial set of known protein sequences was used to search for similar proteins that served for initial pHMMs building. These were refined with additional sequences and rebuilt from improved alignments. NCBI proteins – a database of all the proteins from the finished microbial genomes as described Methods.

The construction of pHMMs [28], [29] was based on a two-step approach (Figure 1). In the first step, an initial pHMM was generated using the HMMER software package [29]. For this purpose, a set of sequences was collected by applying BLAST [30] searches using known protein sequences of described bacterial laccases (Table 1) as templates. The sequences of the obtained hits were aligned using MUSCLE [39]. The alignment was manually processed to remove sequences without the four copper binding domains and duplicates to avoid bias in the models. Phylogenetic analysis was applied to identify different types of target proteins. For phylogenetic tree reconstruction, the neighbor-joining method with Jukes-Cantor genetic distances was used in MEGA4 [40]. Finally, the initial pHMM was generated for each identified group of bacterial laccase-like proteins using the HMMER3 package.

**Table 1:** List of bacterial enzymes for which laccase activity was demonstrated.

accession	source	type	reference
CAB45586	*Streptomyces coelicolor*	2-domain	[31]
BAB64332	*Streptomyces griseus*		[32]
3G5W	*Nitrosomonas europea*		[33]
2ZWN	*metagenomic library*		[34]
YP_077905	*Bacillus licheniformis* ATCC 14580	3-domain	[35]
NP_388511	*Bacillus subtilis* subsp. subtilis str. 168		[18]
NP_414665	*Escherichia coli* str. K-12 substr. MG1655		[36]
AY228142	*Bacillus halodurans*		[22]
YP_005339	*Thermus thermophilus* HB27		[21]
EF507879	*Streptomyces cyaneus* strain CECT3335		[37]
AB092576	*Streptomyces lavendulae*		[20]
AF184209	*Marinomonas mediterranea*		[38]

These sequences were used for BLAST search against the NCBI NR database to retrieve sets of sequences for initial building of profile Hidden Markov Models of bacterial laccases.

In the second step, the pHMMs for the target sequences were retrained. Firstly, the initial pHMMs were applied to search for similar sequences in the pool of protein sequences from microbial genomes stored in the NCBI protein database (described in the following section). Then the sequences were aligned using MUSCLE. Lastly, final pHMMs were constructed for the five identified types of laccases based on the modified version of the alignment. The models cover a large portion of the proteins spanning all four copper-binding domains. The five pHMMs are available as supporting information (Figure S1, S2, S3, S4, S5).

### The databases – genomes and metagenomes

For the pHMM searches, several public databases were used. For the generation and testing of the pHMMs, NCBI proteins and draft proteins were used. The NCBI protein database consisting of 3,819,638 proteins was obtained from a set of 1,216 complete microbial genomes available from the NCBI (genomes – *Prokaryota* database) in September 2010. The organisms belonged to *Bacteria* (89%), *Archaea* (7%) and viruses (4%) and represented 802 different genera. 418 organisms had 1 or more plasmids (a total of 937 plasmids were included in the database). The draft proteins from NCBI is a database of 3,602,197 proteins. The proteins were obtained from 995 draft microbial genomes (apart from one viral and six archaeal genomes, all were bacterial), belonging to 517 genera. It was not distinguished between chromosomes and plasmids as the contigs were not annotated to allow this distinction.

Four different metagenome datasets were used as databases for the pHMM-based search. The metagenome obtained from a biogas plant consisted of “biogas” data contained 1,963,716 nucleotide reads [41], [42]. The sequences were obtained by sequencing on the GS FLX and Titanium platforms and assembled using the Newbler software resulting in 36,483 contigs, which were translated in six reading frames. The “termite metagenome” was a set of 82,789 proteins from the hindgut microbiome of the termite *Nasutitermes* sp. [43] obtained from the IMG/M database. Finally, the “cow rumen” consisted of 2,547,270 proteins from the cow rumen metagenome [44] obtained from the IMG/M database. The dataset of the Global Ocean Survey comprised of 12,672,518 sequences that were retrieved from the CAMERA [45] portal and translated in six reading frames.

### Analyses of microbial genomes

pHMMs were applied to search for bacterial laccase-like sequences in 2,211 microbial genomes organized in two databases as described in the preceding section. Positive hits were aligned using ClustalW. The alignments were manually proof-read and filtered: the sequences without four copper-binding regions were removed. The taxonomic affiliation for the sequences in the final alignment was obtained using the NCBI taxonomy database. The web-based application Phobius [46] was used for the identification of transmembrane segments and signal peptides in the laccase sequences. Several custom-made scripts in Perl, Python and R programming languages were used for the processing and analyses of the data.

### Analyses of metagenomes

The pHMMs were applied to the four metagenomic datasets described in section 2.2. The translated sequences were matched against each pHMM and those matching at least one copper-binding domain were aligned to the corresponding model using the HMMER software package. The alignments were manually processed and non-matching sequences deleted. Finally, the sequences were grouped according to their origin and taxonomically assigned using the lowest common ancestor approach: (1) Each aligned sequence read was compared against the NCBI genomes database using BLAST and an e-value cutoff of e-30. (2) Hits with a bit score above 90% of the best bit score were collected. (3) The lowest common ancestor was calculated for the taxonomies of the selected hits and assigned to the read. The reads for which the lowest common ancestor was identified using this 3-step approach, were aligned to the corresponding model using the HMMER package and manually verified.

### Identification of putative horizontal gene transfer (HGT) events

A parametric method [47] was implemented for a rapid detection of putative HGT events. The algorithm consisted of three steps. (i) For each laccase sequence in the input file, the parent genome was downloaded and the genomic signature was calculated using a 5 kb sliding window with a step of 500 bp as described in [47]. (ii) The distance of each local signature from the average signature was calculated and plotted for a region of ±200 kbp around the locus of the putative laccase sequence. (iii) The figures were then examined by eye to select those where the position of the laccase and a stretch of unusual genomic signature overlapped. These were the putative HGT events; they were additionally examined using BLAST for the presence of other HGT-indications (such as phage integrases or insertion sequences) and to list the genes that had putatively been transferred along with the laccase gene.

## Results

### Identification, diversity and distribution of bacterial laccase-like genes in the genome database entries

A thorough bioinformatics survey of draft and completed bacterial genomes was performed to extensively search for bacterial laccase-like genes. A two-step approach (Figure 1) using pHMMs instead of simple BLAST searches was chosen, allowing the identification of distantly related sequences. In case of two-domain enzymes, two groups were defined corresponding to type B and type C multicopper oxidases as proposed by Nakamura et al. [27]. One profile HMM was deduced for each of these two groups, and the completed and draft bacterial genomes were searched exhaustively by using these new pHMM models. Altogether 221 sequences were obtained (Table 2). More difficulties arose when addressing the diversity of three-domain laccases since many highly diverse sequences were retrieved by searching with the initial profile HMM. Finally, three models were built for the three-domain bacterial laccases. In total, 1019 sequences were retrieved for this laccase type (Table 2).

**Table 2:** The number of sequences retrieved with the newly defined pHMMs when searching the databases of completed and draft genomes.

		No. of genes found		No. (%) of
type	model name	total^a^	unique^b^	signal peptides
two-domain	typeC2D	63	63	40 (63.5)
	typeB2D	158	158	127 (80.4)
three-domain	small3D	822	355	303 (85.4)
	big3D	308	159	118 (74.2)
	cot3D	200	38	26 (68.4)
	more than one model^c^	/	467	329 (70.4)
		sum	1240	943 (76.0)

a No. of genes retrieved with the model.

b No. of genes not retrieved with any other model.

c No. of genes retrieved with more that one model.

The pHMMs and the sequences are available as supplementary information (Figure S1, S2, S3, S4, S5, S6). Signal peptides were identified using the on-line version of Phobius [46].

Signal peptides and transmembrane segments in the obtained laccase amino acid sequences were identified using Phobius [46]. Three quarters of the enzymes harbored putative signal peptides (Table 2), indicating that the majority of the bacterial laccases may be exported out of the cytoplasm which is in contrast to the current knowledge [16].

In total, 1240 genes for laccase-like enzymes have been found in 807 different microorganisms (36% of 2211 organisms included in the study). The sequences are available as supplementary information (Figure S6). In 252 organisms more than one laccase gene was identified (58 organisms encoded 3 genes, 18 encoded 4 genes, 16 had 5 genes and 7 harbored more than 5 laccase-like genes). The highest number of putative laccase genes was identified in *Xanthobacter autotrophicus* Py2, where three out of the 10 laccase genes were encoded on a plasmid and both two- and three-domain enzymes were present on the chromosome and the plasmid. Both *Sulfitobacter* sp. NAS-14.1 and *Sorangium cellulosum* So ce 56 had eight genes in their chromosomes, with one two-domain laccase in each genome while the others were three-domain enzymes.

**Figure 2:**
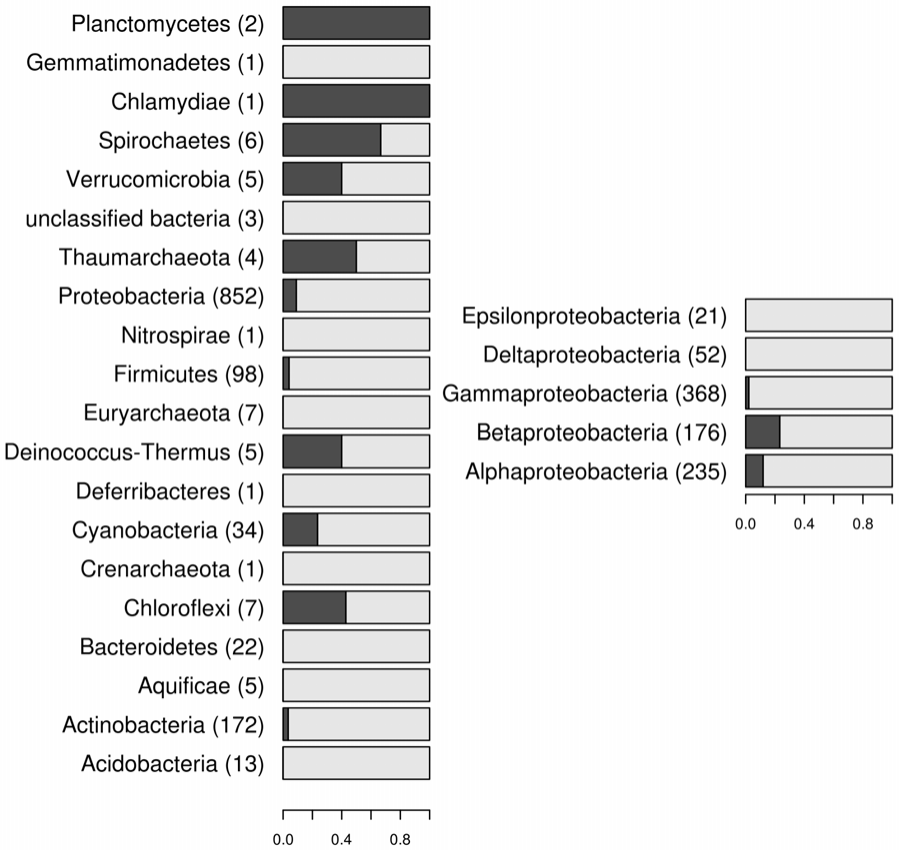
Proportions of two-domain (black) and three-domain (grey) laccases in different phyla (left) and classes of Proteobacteria (right). The numbers in brackets represent the total number of laccase genes found in each taxon.

**Figure 3:**
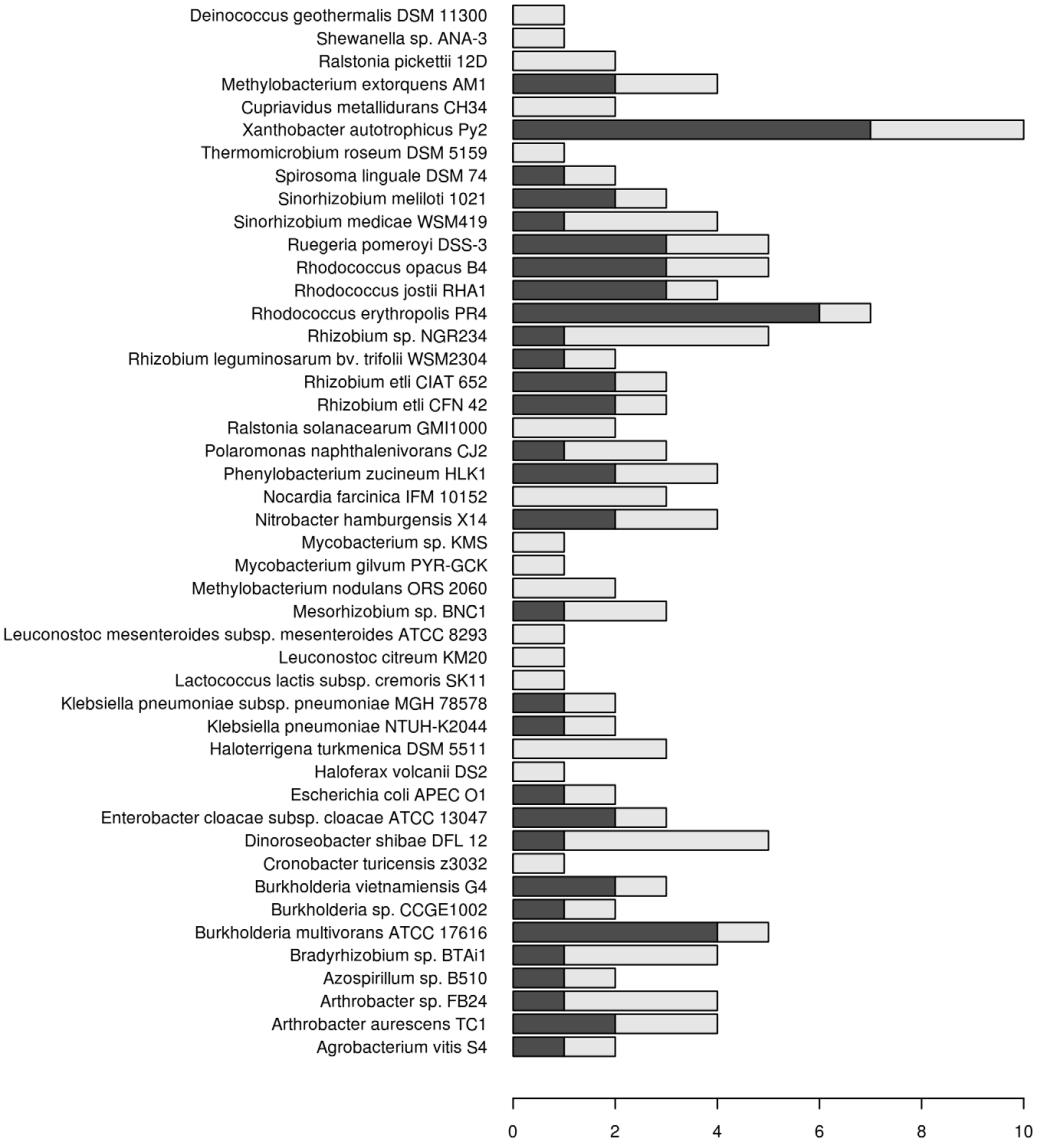
List of species encoding laccase genes and possessing plasmids in their genomes. The bars represent the number of laccase genes in the genome (black) and the number of laccase genes on plasmids (gray). The length of the bar shows the total number of genes for each organism.

Several phyla are represented with very few sequences while in other groups many laccase genes were retrieved (Figure 2). For example, as many as 368 sequences in the final dataset were affiliated to *Gammaproteobacteria*. However, only 14 of these (<4%) were two-domain laccases, which were completely absent in the groups *Deltaproteobacteria* and *Epsilonproteobacteria*. Only few two-domain laccases were identified in *Actinobacteria*, which is surprising, since two-domain laccases had predominantly been discovered in *Streptomyces* [31], [32]. While *Acidobacteria* and *Bacteroidetes* seemed to lack two-domain laccases, only two-domain laccases were found in *Planctomycetes* (admittedly only two sequences in ten sequenced genomes from this phylum). Moreover, 34 genes were identified in *Cyanobacteria*. Interestingly, all 8 two-domain laccases in *Cyanobacteria* belonged to type C (Figure 2). In finished genome projects, it was possible to obtain information on the location of the genes (whether on the chromosome or a plasmid). From the 749 genes identified in the finished genomes, 76 genes (52 for three-domain and 24 for two-domain laccases) were encoded on plasmids originating from 46 different organisms (Figure 3). One third of these (34%) were associated with various *Rhizobiales* species that usually had multiple genes for laccases in their genomes. *Enterobacteria,* on the other hand, typically contained just a single gene for a three-domain laccase, except for eleven enterobacterial strains that carried two genes for laccase-like enzymes. Here, the second gene was usually encoded on a plasmid (Figure 3), e.g. *Klebsiella pneumoniae* NTUH-K20 plasmid pK2044 (NC_006625.1, protein YP_001687946.1) and *Escherichia coli* APEC O1 plasmid pAPEC-O1-R (NC_009838.1, protein YP_001481473.1). In contrast, in some organisms (e.g. species of *Mycobacterium*, *Ralstonia* and *Leuconostoc*) laccase-like genes were identified only on plasmids (Figure 3).

### Bacterial laccase-like sequences in metagenomic datasets

Only 62 low-scoring hits were obtained with the new pHMMs when searching the metagenome originating from a biogas-producing microbial community [41], [42]. BLAST searches affiliated most sequences to the archeon *Methanoculleus* and different *Clostridium/Bacillus* species. Few hits were found when searching the metagenomes obtained from anaerobic microbial communities in termite and cow digestive systems, although these communities actively degrade plant biomass. The hits were not significant and none covered any of the copper-binding regions within laccase sequences.

**Figure 4:**
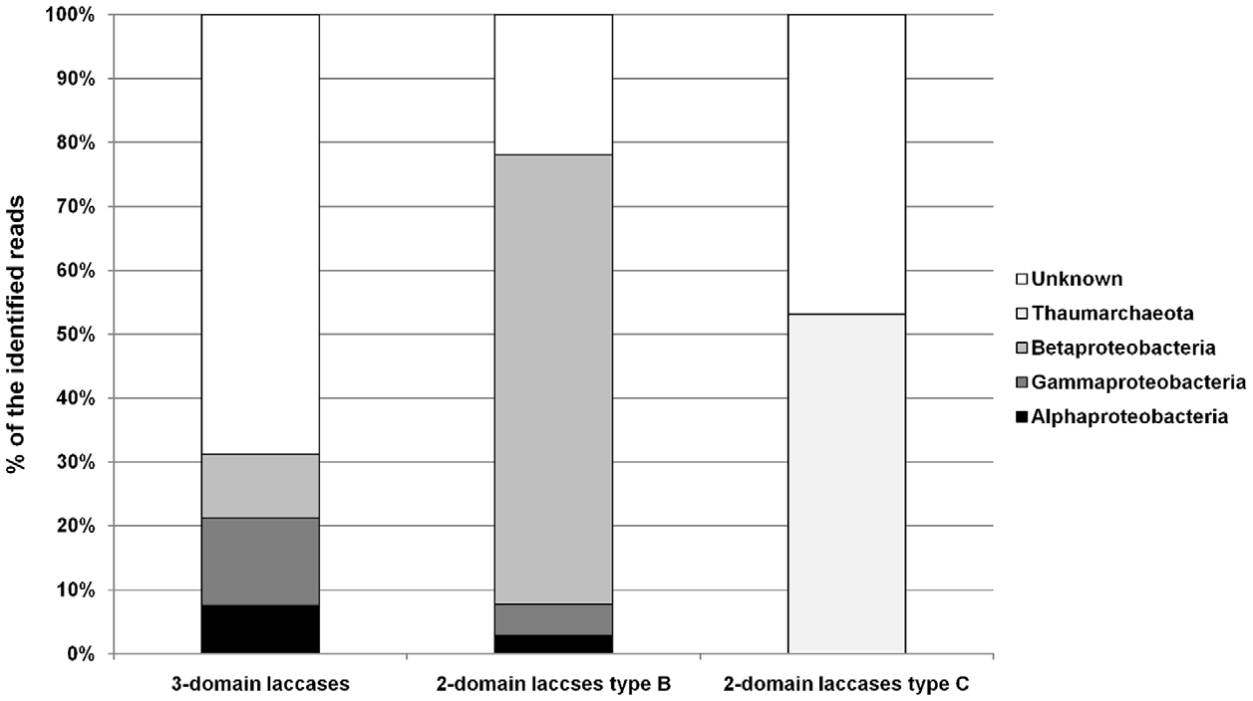
Taxonomic affiliation of sequences encoding laccases-like proteins in the Global Ocean Survey data. Proportions are shown for the total of 847, 245 and 32 sequences aligning well to the pHMMs of the three-domain laccases, type B two-domain laccases and type C two-domain laccases, respectively.

The pHMM based search of the Global Ocean Survey data, on the other hand, retrieved numerous hits for prokaryotic laccases. In total, 277 and 847 sequences showed similarities to two-domain and three-domain laccase-like sequences, respectively, aligning neatly to the copper-binding regions of the models. However, only 33% of the putative three-domain laccases could be affiliated to *Bacteria* using the lowest common ancestor approach; the rest did not resemble any sequence in public databases, indicating considerable novelty. The majority (97%) of the classifiable sequences were assigned to *Proteobacteria* while the remaining were assigned to *Cyanobacteria*, *Bacteroidetes* and *Actinobacteria* (Figure 4). At the genus level, most sequences were assigned to *Burkholderia* and *Shewanella.*

For the two-domain laccases, all four existing copper-binding regions were identified in some environmental gene tags (EGTs). The taxonomic affiliations of the sequences of the type B and type C two-domain laccases were analyzed separately (Figure 4). In total, only 53% of the type C sequences could be affiliated to *Archaea* (*Thaumarchaeota – Nitrosopumilus*), while no similar sequences could be found in the public databases for the remaining half of the dataset. For the type B two-domain laccase sequences, 87% were assigned to *Bacteria*. *Betaproteobacteria* were the dominant class (90% of all classifiable reads), mostly represented by the *Burkholderia*-associated sequences (84%) (Figure 4).

The origin of the identified laccase-like sequences was analyzed in more detail. Most of the three-domain laccase-like sequences (30%) were found in the Sargasso Sea at the station 11 where the sea temperature at the time of sampling was 20.5°C, the chlorophyll density was 0.17 µg/kg and the salinity was 36.7 ppt. 14% of the reads encoding three-domain laccase like enzymes were obtained from the sample from a Galapagos Islands sample, taken at Punta Cormorant. This location featured a high salinity of 63.4 ppt and a sea temperature of 37.6°C. The two-domain laccases were mainly obtained from the Sargasso Sea station 11 (52%) and from Sargasso Sea station 3 (7%).

### Horizontal gene transfer of laccase-like sequences identified in microbial genomes

Identification of potentially horizontally transferred laccase genes was based on tetraoligonucleotide frequencies or genomic signatures [47]. Local genomic signatures covering laccase genes were compared to average signatures of respective genomes. If the local signatures were significantly different, the corresponding genes were considered to have been acquired *via* horizontal gene transfer (HGT) and the fragment was further examined for the presence of other indicators of HGT, such as remainders of insertion sequences, transposons or phages. Possible other open reading frames on the fragment were annotated to elucidate the genetic potential of the fragment for the organism. More than 40 examples representing putative HGT events were found (not all examined in further detail), and four examples are described below.

In *Nitrosococcus watsoni* C-113, a short fragment of less than 5 kbp was identified as having been horizontally transferred, carrying a putative two-domain laccase (YP_003760803) and an outer membrane efflux protein (YP_003760804) gene. There were also the remnants of genes similar to a resolvase and a transposase, again indicating past horizontal gene transfer of the fragment.

Of the 34 *Yersinia* strains included in the study, more than one gene for a laccase-like enzyme was found only in the draft genome of *Yersinia mollaretii* ATCC 43969. At the beginning of the putatively horizontally transferred fragment, remnants of a phage integrase gene were detected. In addition, several putative open reading frames could be predicted but most of them were small and did not resemble any known sequences in the database. However, four open reading frames other than the ORF for the putative three-domain laccase are homologous to database entries. These encode a protein with a beta-lactamase domain, an ABC transporter, and two cytochrome c family proteins.

Out of 40 genomes from the genus *Haemophilus* included in our study, only one strain contained two laccase-like genes. These two genes were identified on a common genomic island that has a markedly different signature than the rest of the genome. Close to the two laccase genes was a gene encoding a heavy metal translocating ATPase and a longer stretch (2500bp) of DNA with a homology to plasmids and transposons of various organisms, harboring genes for tetracycline resistance. The end of the fragment showed a similarity to putative integrase genes as revealed by BLASTn.

In *Geobacter sulfurreducens*, the putative horizontally transferred fragment encoded a ferrous ion transport protein, a set of CRISPR-associated genes (known for their mobility through HGT) and a laccase gene.

## Discussion

The aim of this study was to identify laccase-like genes in published bacterial genomes and metagenome datasets. Five new profile Hidden Markov Models (pHMM) were developed for two-domain and three-domain laccases. Such probabilistic models of protein families are commonly used in the analysis of high-throughput sequencing data [48]. The main advantage of a pHMM-based approach is the high accuracy in detecting conserved domains compared to other methods such as BLAST.

Specific pHMMs were developed for type B and type C two-domain laccases that were previously identified [27]. These models are particularly important since two-domain multicopper oxidases could not be efficiently discovered with the existing models for fungal laccases. Several genes for the type A two-domain laccases have also been identified with the initial BLAST-based searches. However, these originated solely from *Archaea* such as *Halogeometricum borinquense* DSM 11551 (3 genes), *Haloterrigena turkmenica* DSM 5511 (2 genes), *Haloarcula marismortui* ATCC 43049 (2 genes), *Halobacterium sp.* NRC-1 (this gene was previously identified by [27]), and were thus excluded from further analysis.

The diversity of three-domain bacterial laccases, which are similar to those in fungi, was unexpectedly high; three models were constructed to capture most of the variability in amino acid sequences and lengths of the predicted proteins. Based on the sizes of individual domains and whole proteins, two major groups were identified: a larger group of enzymes (81% of the three-domain laccases) with the well-known representatives such as CotA from *Bacillus subtilis* (identified with the pHMM named small3D), and a smaller group (16% of the three-domain laccases) including considerably larger proteins that to our knowledge has no characterized representatives (these were retrieved with the pHMM named big3D). Bacteria of the genera *Pseudomonas*, *Geobacter, Xanthobacter* and *Acinetobacter* were found to possess laccases belonging to this second group. However, the diversity within these two groups, e.g. the diversity in the copper-binding regions, was also notable. Laccases from smaller taxa of closely related bacteria with few copies in each genome (e.g. *Enterobacteria*) clustered together in phylogenetic trees (data not shown) while the clustering of laccases from some phyla was not consistent with the 16S rRNA phylogeny. Notably, laccases from *Firmicutes* (low-GC) and *Actinobacteria* (high-GC) formed several mixed clusters that often included laccases from *Proteobacteria* (data not shown).

The high level of diversity within groups of related bacteria suggested two mutually non-excluding explanations. Firstly, it is evident from our data that there are several groups of bacterial laccases in terms of protein structure and, we speculate, also in terms of physiological function (substrate utilization, pigment formation, stress resistance and others yet to be discovered). Further members of these subgroups of bacterial laccases may be identified in due time on the basis of studies such as this one. Secondly, horizontal gene transfer may offer some explanations as to why laccases from the same organism can be so diverse. The present study provides some evidence that certain laccase genes were probably acquired *via* horizontal gene transfer, either alone or together with other important genes such as antibiotic resistance genes.

It is important to note that the microbial genome databases are extremely biased towards certain organisms. Although measures have been undertaken to help relieve this issue and many genomes of organisms from scarcely represented phyla are being added [49], several major bacterial groups are still represented with very few sequenced genomes. This bias is reflected in our data as several phyla were represented with less than 10 sequences (Figure 2) while some groups were large (for example, as many as 368 sequences in the final dataset were affiliated to *Gammaproteobacteria* due to the pronounced bias towards *Enterobacteria*). This fact makes it difficult to infer general conclusions about the presence or absence of genes for certain types of laccases in a particular group of organisms. Moreover, finding genes encoding laccase-like enzymes does not necessarily mean that the organism has laccase activity at its disposal.

Still, the results presented here indicate considerable diversity of laccases in bacteria and question some of the current views of bacterial laccases. Most notably, based on the presence of signal peptides around 76% of the putative proteins identified in this study appear to be secreted from the cytoplasm. Moreover, the genes for laccase-like enzymes were found in anaerobic organisms. Corresponding enzymes almost invariably had signal peptides indicating that they may be active in a more aerobic environment away from the cells. This is a possible scenario for some soil bacteria (e.g. *Geobacter, Clostridium*) but quite unlikely for microbes living in the anaerobic digestive systems of herbivores. This is probably the reason why very few laccases were found in metagenomes derived from these habitats – organisms living in digestive tracts probably use other enzymes for the breakdown of plant (poly)phenolics, such as diverse peroxidases. There is evidence that some bacterial laccases are indeed involved in lignin degradation [19], while others may carry out functions such as pigment formation, as shown for the CotA laccase from *Bacillus subtilis* [18]. Moreover, many autotrophs have laccases, for example *Cyanobacteria* (34 genes in 23 organisms) and nitrifying bacteria (28 genes in 9 organisms of the genera *Nitrosococcus, Nitrosomonas and Nitrobacter*).

As reviewed in the introduction, bacterial laccases may also be interesting for biotechnological applications. However, there have only been a few attempts to verify this in practice. Notably, the CotA laccase was able to decolorize a variety of structurally different synthetic dyes at alkaline pH and in the absence of redox mediators [50]. Azo-dyes have been degraded with an unusual two-domain laccase from *Streptomyces* that is active in a dimeric form and exhibits high thermo- and pH-stability [51]. It has been shown that xenobiotics increase the activity of the laccase from a *Gammaproteobacterium*, which may indicate the protective role of laccases against mutagens, xenobiotics and agrochemicals [52]. There is a growing body of evidence that *Bacteria* can degrade lignin [19] and that laccases are important in this process, either acting alone or together with other enzymes such as extracellular peroxidases [4]. Bacterial laccases certainly will have to be taken into account in future enzyme cocktails for lignin degradation or diverse environmental applications.

The present study can help to identify potential novel sources of laccases. These are the organisms with multiple genes for laccases; for example, *Rhodococcus erythropolis* PR4 had one gene on a plasmid and six other genes encoded on the chromosome. Bacteria of the genus *Rhodococcus* were shown to degrade different types of lignin in the absence of hydrogen peroxide, indicating that laccases may be involved in the process [19]. *Rhodococci* are known to be potent degraders of polychlorinated biphenyls (PCBs) and since biphenyls occur naturally due to lignin degradation, it has been speculated that the enzymes responsible for PCB degradation had originally been involved in lignin degradation [19]. In this study, 26 genes for laccase-like enzymes could be identified in only five different species of the genus *Rhodococcus*.

Salt- and pH-tolerant laccases are desired for industrial applications and one laccase exhibiting such properties has been described in *Bacillus halodurans* [22]. A novel laccase, found using a metagenomic approach, was extremely halotolerant (up to 1M NaCl) and pH-stable and could degrade several synthetic dyes, some of them even in the absence of mediators [53]. Many alkaliphilic bacteria are currently being sequenced by the Joint Genome Institute (e. g. *Heliothrix oregonensis, Thioalkalivibrio thiocyanoxidans, Thioalkalimicrobium cyclicum*) and it will be interesting to search their genomes for laccase genes once they become available. However, some genes for laccase-like enzymes have been discovered in the alkaliphiles available at the time of this study, for example in *Oceanobacillus iheyensis* (*Firmicutes*) and *Thioalkalivibrio sp.* (three genes in two strains of these *Gammaproteobacteria*). These organisms may also be important sources of novel enzymes with desired properties.

Recently, the Laccase Engineering Database (LccED) was launched with an ambition to collect and manage molecular data regarding laccases and related multicopper oxidases from all domains of life [54]. Over 2200 proteins were collected in the LccED, and laccases from fungi and plants predominated. Their dataset of bacterial laccases overlapped with ours to a large extent (up to 70% of the sequences). Their collection was richer for the environmental sequences but also contained sequences which appeared not to be laccases as they contained no copper-binding domains (e.g. CAA78165.1). Conversely, our search retrieved several hundred new sequences. One of the distinctive findings of our study was to identify 220 genes for the two-domain laccase enzymes, while the LccED database listed less than 20. LccED is certainly a valuable resource that may be further enriched with sequences from studies such as the present one. By facilitating the access to taxonomic information and by enabling batch assignments to the proposed protein families, the LccED could enable the researchers to elegantly investigate topics similar to the ones addressed here.

## Conclusions

In the present study, an enormous amount of sequence data was made accessible to study an increasingly important group of enzymes. Both two- and three-domain laccases were retrieved. The results supported our hypothesis that the genes for laccases were widely distributed among virtually all bacterial phyla. We showed that the localization of bacterial laccases may not be restricted to the cytoplasm and that they may be rather mobile. Moreover, these genes abounded in anaerobic organisms and autotrophs, and we pointed to some interesting organisms that could be exploited for their laccases. Admittedly, the majority of the putative enzymes discussed in this paper still need to be experimentally verified. However, elucidation of the wide distribution and enormous diversity of bacterial genes for laccase-like enzymes will undoubtedly increase scientific interest in this emerging field.

## Supporting Information

Figure S1 **Profile Hidden Markov Model for type C two-domain laccases.** The pHMM file “typeC2D.hmm“ was generated with HMMER software (28).
(HMM)

Figure S2 **Profile Hidden Markov Model for type B two-domain laccases.** The pHMM file “typeB2D.hmm“ was generated with HMMER software (28).
(HMM)

Figure S3 **Profile Hidden Markov Model for three-domain laccases.** The pHMM file “small3D.hmm“ was generated with HMMER software (28).
(HMM)

Figure S4 **Profile Hidden Markov Model for three-domain laccases.** The pHMM file “big3D.hmm“ was generated with HMMER software (28).
(HMM)

Figure S5 **Profile Hidden Markov Model for three-domain laccases.** The pHMM file “cot3D.hmm“ was generated with HMMER software (28).
(HMM)

Figure S6 **Amino acid sequences of 1240 putative laccases identified in the present study.** The sequences are in fasta format, each sequence has the accession number and the name of the source organism in the description.
(FAS)

## References

[1] Rodríguez-Rodríguez CE, Jelic A, Llorca M, Farré M, Caminal G (2011). Solid-phase treatment with the fungus *Trametes versicolor* substantially reduces pharmaceutical concentrations and toxicity from sewage sludge.. Bioresource Technol.

[2] Auriol M, Filali-Meknassi Y, Tyagi RD, Adams CD (2007). Laccase-catalyzed conversion of natural and synthetic hormones from a municipal wastewater.. Water Res.

[3] Theuerl S, Buscot F (2010). Laccases: toward disentangling their diversity and functions in relation to soil organic matter cycling.. Biol and Fert Soils.

[4] Bugg TDH, Ahmad M, Hardiman EM, Singh R (2011). The emerging role for bacteria in lignin degradation and bio-product formation..

[5] Couto SR, Herrera JLT (2006). Industrial and biotechnological applications of laccases: A review.. Biotechnol Adv.

[6] Widsten P, Kandelbauer A (2008). Laccase applications in the forest products industry: A review.. Enzyme Microb Tech.

[7] Singh Arora D, Kumar Sharma R (2010). Ligninolytic fungal laccases and their biotechnological applications.. Appl Biochem Biotech.

[8] Claus H (2003). Laccases and their occurrence in prokaryotes.. Arch Microbiol.

[9] Luis P, Walther G, Kellner H, Martin F, Buscot F (2004). Diversity of laccase genes from basidiomycetes in a forest soil.. Soil Biol Biochem.

[10] Kellner H, Luis P, Zimdars B, Kiesel B, Buscot F (2008). Diversity of bacterial laccase-like multicopper oxidase genes in forest and grassland Cambisol soil samples.. Soil Biol Biochem.

[11] Ausec L, van Elsas JD, Mandic-Mulec I (2011). Two- and three-domain bacterial laccase-like genes are present in drained peat soils.. Soil Biol Biochem.

[12] Hoegger PJ, Kilaru S, James TY, Thacker JR, Kues U (2006). Phylogenetic comparison and classification of laccase and related multicopper oxidase protein sequences.. FEBS J.

[13] Baldrian P (2006). Fungal laccases – occurrence and properties. FEMS Microbiol. Rev..

[14] Givaudan A, Effosse A, Faure D, Potier P, Bouillant ML (1993). Polyphenol oxidase in *Azospirillum lipoferum* isolated from rice rhizosphere: Evidence for laccase activity in non-motile strains of *Azospirillum lipoferum*.. FEMS Microbiol Lett.

[15] Alexandre G, Zhulin IB (2000). Laccases are widespread in bacteria.. Trends Biotechnol.

[16] Sharma P, Goel R, Capalash N (2007). Bacterial laccases.. World J Microb Biot.

[17] Kilaru S, Hoegger P, Kües U (2006). The laccase multi-gene family in *Coprinopsis cinerea* has seventeen different members that divide into two distinct subfamilies.. Curr Genet.

[18] Martins LO, Soares CM, Pereira MM, Teixeira M, Costa T (2002). Molecular and biochemical characterization of a highly stable bacterial laccase that occurs as a structural component of the *Bacillus subtilis* endospore coat.. J Biol Chem.

[19] Ahmad M, Taylor CR, Pink D, Burton K, Eastwood D (2010). Development of novel assays for lignin degradation: comparative analysis of bacterial and fungal lignin degraders.. Mol BioSyst.

[20] Suzuki T, Endo K, Ito M, Tsujibo H, Miyamoto K (2003). A thermostable laccase from *Streptomyces lavendulae* REN-7: purification, characterization, nucleotide sequence, and expression.. Biosci Biotechnol Biochem.

[21] Miyazaki K (2005). A hyperthermophilic laccase from *Thermus thermophilus* HB27.. Extremophiles.

[22] Ruijssenaars HJ, Hartmans S (2004). A cloned *Bacillus halodurans* multicopper oxidase exhibiting alkaline laccase activity.. Appl Microbiol Biotechnol.

[23] Singh G, Capalash N, Goel R, Sharma P (2007). A pH-stable laccase from alkali-tolerant γ-proteobacterium JB: Purification, characterization and indigo carmine degradation.. Enzyme Microb Tech.

[24] Niladevi KN, Jacob N, Prema P (2008). Evidence for a halotolerant-alkaline laccase in *Streptomyces psammoticus*: Purification and characterization.. Process Biochem.

[25] Dube E, Shareck F, Hurtubise Y, Daneault C, Beauregard M (2008). Homologous cloning, expression, and characterisation of a laccase from *Streptomyces coelicolor* and enzymatic decolourisation of an indigo dye.. Appl Microbiol Biot.

[26] Ye M, Li G, Liang W, Liu YH (2010). Molecular cloning and characterization of a novel metagenome-derived multicopper oxidase with alkaline laccase activity and highly soluble expression.. Appl Microbiol Biot.

[27] Nakamura K, Kawabata T, Yura K, Go N (2003). Novel types of two-domain multi-copper oxidases: possible missing links in the evolution.. FEBS Letters.

[28] Durbin R, Eddy S, Krogh A, Mitchinson G (1998). Biological sequence analysis: Probabilistic models of proteins and nucleic acids..

[29] Eddy SR (1998). Profile hidden Markov models.. Bioinformatics.

[30] Altschul SF, Gish W, Miller W, Myers EW, Lipman DJ (1990). Basic local alignment search tool.. J Mol Biol.

[31] Machczynski MC, Vijgenboom E, Samyn B, Canters GW (2004). Characterization of SLAC: A small laccase from *Streptomyces coelicolor* with unprecedented activity.. Protein Sci..

[32] Endo K, Hayashi Y, Hibi T, Hosono K, Beppu T (2003). Enzymological characterization of EpoA, a laccase-like phenol oxidase produced by *Streptomyces griseus*.. J Biochem.

[33] Lawton TJ, Sayavedra-Soto LA, Arp DJ, Rosenzweig AC (2009). Crystal structure of a two-domain multicopper oxidase: implications for the evolution of multicopper blue proteins.. J Biol Chem.

[34] Komori H, Miyazaki K, Higuchi Y (2009). X-ray structure of a two-domain type laccase: a missing link in the evolution of multi-copper proteins.. FEBS Lett.

[35] Koschorreck K, Richter SM, Ene AB, Roduner E, Schmid RD (2008). Cloning and characterization of a new laccase from *Bacillus licheniformis* catalyzing dimerization of phenolic acids.. Appl Microbiol Biotechnol.

[36] Roberts SA, Wildner GF, Grass G, Weichsel A, Ambrus A (2003). A labile regulatory copper ion lies near the T1 copper site in the multicopper oxidase CueO.. J Biol Chem.

[37] Arias ME, Arenas M, Rodriguez J, Soliveri J, Ball AS (2003). Kraft pulp biobleaching and mediated oxidation of a nonphenolic substrate by laccase from *Streptomyces cyaneus* CECT 3335.. Appl Environ Microbiol.

[38] Sanchez-Amat A, Lucas-Elío P, Fernández E, García-Borrón JC, Solano F (2001). Molecular cloning and functional characterization of a unique multipotent polyphenol oxidase from *Marinomonas mediterranea*.. Biochim Biophys Acta.

[39] Edgar RC (2004). MUSCLE: multiple sequence alignment with high accuracy and high throughput.. Nucleic Acids Res.

[40] Tamura K, Dudley J, Nei M, Kumar S (2007). MEGA4: Molecular Evolutionary Genetics Analysis (MEGA) software version 4.0.. Mol Biol Evol.

[41] Jaenicke S, Ander C, Bekel T, Bisdorf R, Dröge M (2011). Comparative and joint analysis of two metagenomic datasets from a biogas fermenter obtained by 454-pyrosequencing.. PLoS One.

[42] Schlüter A, Bekel T, Diaz NN, Dondrup M, Eichenlaub R (2008). The metagenome of a biogas-producing microbial community of a production-scale biogas plant fermenter analysed by the 454-pyrosequencing technology.. J Biotechnol.

[43] Warnecke F, Luginbühl P, Ivanova N, Ghassemian M, Richardson TH (2007). Metagenomic and functional analysis of hindgut microbiota of a wood-feeding higher termite.. Nature.

[44] Hess M, Sczyrba A, Egan R, Kim T-W, Chokhawala H (2011). Metagenomic discovery of biomass-degrading genes and genomes from cow rumen.. Science.

[45] Seshadri R, Kravitz SA, Smarr L, Gilna P, Frazier M (2007). CAMERA: a community resource for metagenomics.. PLoS Biol.

[46] Käll L, Krogh A, Sonnhammer EL (2007). Advantages of combined transmembrane topology and signal peptide prediction - the Phobius web server.. Nucleic Acids Res.

[47] Dufraigne C, Fertil B, Lespinats S, Giron A, Deschavanne P (2005). Detection and characterization of horizontal transfers in prokaryotes using genomic signature.. Nucleic Acids Res.

[48] Krause L, Diaz NN, Edwards RA, Gartemann K-H, Krömeke H (2008). Taxonomic composition and gene content of a methane-producing microbial community isolated from a biogas reactor.. J Biotechnol.

[49] Wu D, Hugenholtz P, Mavromatis K, Pukall R, Dalin E (2009). A phylogeny-driven genomic encyclopaedia of Bacteria and Archaea.. Nature.

[50] Pereira L, Coelho AV, Viegas CA, Santos MC, Robalo MP, Martins LO (2009). Enzymatic biotransformation of the azo dye Sudan Orange G with bacterial CotA-laccase.. J Biotechnol.

[51] Molina-Guijarro JM, Perez J, Munoz-Dorado J, Guillen F, Moya R (2009). Detoxification of azo dyes by a novel pH-versatile, salt-resistant laccase from *Streptomyces ipomoea*.. Int Microbiol.

[52] Singh G, Batish M, Sharma P, Capalash N (2009). Xenobiotics enhance laccase activity in alkali-tolerant γ-proteobacterium JB.. Braz. J. Microbiol..

[53] Fang Z, Li T, Wang Q, Zhang X, Peng H (2011). A bacterial laccase from marine microbial metagenome exhibiting chloride tolerance and dye decolorization ability.. Appl Microbiol Biotechnol.

[54] Sirim D, Wagner F, Wang L, Schmid RD, Pleiss J (2011). The laccase engeneering database: a classification and analysis system for laccases and related multicopper oxidases.. http://dx.doi.org/10.1093/database/bar006.

