# Correction: Bioinformatic Analysis Reveals High Diversity of Bacterial Genes for Laccase-Like Enzymes

**DOI:** 10.1371/annotation/2c1232ba-94f0-4613-8486-0f430b1b47ed

**Published:** 2012-01-04

**Authors:** Luka Ausec, Martha Zakrzewski, Alexander Goesmann, Andreas Schlüter, Ines Mandic-Mulec

There was an error in the author attributions indicated in the byline. Luka Ausec and Martha Zakrzewski are co-first authors and contributed equally to this work. 

